# Variant-specific effects define the phenotypic spectrum of *HNRNPH2-*associated neurodevelopmental disorders in males

**DOI:** 10.1007/s00439-021-02412-x

**Published:** 2021-12-14

**Authors:** Hans-Jürgen Kreienkamp, Matias Wagner, Heike Weigand, Allyn McConkie-Rossell, Marie McDonald, Boris Keren, Cyril Mignot, Julie Gauthier, Jean-François Soucy, Jacques L. Michaud, Meghan Dumas, Rosemarie Smith, Ulrike Löbel, Maja Hempel, Christian Kubisch, Jonas Denecke, Philippe M. Campeau, Jennifer M. Bain, Davor Lessel

**Affiliations:** 1 Institute of Human Genetics, University Medical Center Hamburg-Eppendorf, Martinistrasse 52, 20246 Hamburg, Germany; 2 Institute of Human Genetics, Technical University of Munich, Munich, Germany; 3 Department of Pediatric Neurology, Developmental Medicine and Social Pediatrics, Dr. von Hauner’s Children’s Hospital, University of Munich, Munich, Germany; 4 Division of Medical Genetics, Department of Pediatrics, Duke University, Durham, USA; 5 Département de Génétique, Hôpital La Pitié-Salpêtrière, Assistance Publique-Hôpitaux de Paris, Paris, France; 6 Molecular Diagnostic Laboratory, CHU Sainte-Justine, Montreal, QC Canada; 7 Division of Medical Genetics, Department of Pediatrics, CHU Sainte-Justine and Université de Montréal, Montreal, QC Canada; 8 Division of Genetic, Department of Pediatrics, The Barbara Bush Children’s Hospital, Maine Medical Center, Portland, ME USA; 9 Department of Diagnostic and Interventional Neuroradiology, University Medical Center Hamburg-Eppendorf, Hamburg, Germany; 10 Department of Pediatrics, University Medical Center Eppendorf, Hamburg, Germany; 11 Department of Pediatrics, CHU Sainte-Justine and University of Montreal, Montreal, Canada; 12 Division of Child Neurology, Department of Neurology, Columbia University Irving Medical Center, New York, USA

## Abstract

Bain type of X-linked syndromic intellectual developmental disorder, caused by pathogenic missense variants in *HRNRPH2*, was initially described in six female individuals affected by moderate-to-severe neurodevelopmental delay. Although it was initially postulated that the condition would not be compatible with life in males, several affected male individuals harboring pathogenic variants in *HNRNPH2* have since been documented. However, functional in-vitro analyses of identified variants have not been performed and, therefore, possible genotype–phenotype correlations remain elusive. Here, we present eight male individuals, including a pair of monozygotic twins, harboring pathogenic or likely pathogenic *HNRNPH2* variants. Notably, we present the first individuals harboring nonsense or frameshift variants who, similarly to an individual harboring a de novo p.(Arg29Cys) variant within the first quasi-RNA-recognition motif (qRRM), displayed mild developmental delay, and developed mostly autistic features and/or psychiatric co-morbidities. Additionally, we present two individuals harboring a recurrent de novo p.(Arg114Trp), within the second qRRM, who had a severe neurodevelopmental delay with seizures. Functional characterization of the three most common *HNRNPH2* missense variants revealed dysfunctional nucleocytoplasmic shuttling of proteins harboring the p.(Arg206Gln) and p.(Pro209Leu) variants, located within the nuclear localization signal, whereas proteins with p.(Arg114Trp) showed reduced interaction with members of the large assembly of splicing regulators (LASR). Moreover, RNA-sequencing of primary fibroblasts of the individual harboring the p.(Arg114Trp) revealed substantial alterations in the regulation of alternative splicing along with global transcriptome changes. Thus, we further expand the clinical and variant spectrum in *HNRNPH2*-associated disease in males and provide novel molecular insights suggesting the disorder to be a spliceopathy on the molecular level.

**Supplementary Information:**

The online version contains supplementary material available at 10.1007/s00439-021-02412-x.

## Introduction

Heterogeneous nuclear ribonucleoproteins (hnRNPs) constitute a large group of RNA-binding proteins with multiple roles in RNA metabolism including the regulation of transcription, translation, mRNA stability, mRNA decay, and splicing (Geuens et al. 2016). Currently, there are more than 20 known hnRNPs that were named hnRNPs A–U (Han et al. 2010). Previous studies established pathogenic variants in several genes encoding for hnRNPs as the underlying cause of neurodegenerative and neurodevelopmental disorders. The examples include pathogenic variants in *HNRNPA1* (OMIM #164017) and *HNRNPA2B1* (OMIM #600124) identified in individuals affected by neurodegenerative diseases including amyotrophic lateral sclerosis (Kim et al. 2013). In addition, pathogenic variants in *HNRNPU* (OMIM #602869) (Carvill et al. 2013; Bramswig et al. 2017), *HNRNPK* (OMIM #600712) (Au et al. 2015), *HNRNPH2* (OMIM #300610) (Bain et al. 2016), *HNRNPH1* (OMIM #601035) (Pilch et al. 2018; Reichert et al. 2020), and *HNRNPR* (OMIM #607201) (Duijkers et al. 2019) were identified in individuals affected by various neurodevelopmental disorders. Moreover, a recent large-scale sequencing study additionally implicated a role for *HNRNPAB* (OMIM #602688), *HNRNPD* (OMIM #601324), *HNRNPF* (OMIM #601037), *HNRNPH3* (OMIM #602324), *HNRNPUL1* (OMIM #605800), *HNRNPUL2* (currently no OMIM #), and *HNRNPQ*/*SYNCRIP* (OMIM #616686) in human neurodevelopmental disorders (Gillentine et al. 2021). These data further establish the importance of RNA metabolism in governance of non-dividing neurons and especially the role of the hnRNP gene family in the development and function of complex neuronal circuits.

De novo missense variants in *HNRNPH2* (OMIM #300610) were first identified in six female individuals affected by a neurodevelopmental delay, termed Bain type of X-linked syndromic intellectual developmental disorder (OMIM #300986) (Bain et al. 2016). Initially, it was anticipated that male individuals bearing pathogenic variants in this gene would not be viable (Bain et al. 2016). However, nine males affected by a severe neurodevelopmental delay harboring a pathogenic hemizygous *HNRNPH2* variant have since been reported in the literature (Gillentine et al. 2021; Harmsen et al. 2019; Jepsen et al. 2019; Somashekar et al. 2020; Bain et al. 2021). HNRNPH2 is an RNA-binding protein that specifically recognizes G-tract RNA sequences involved in pre-mRNA processing, both by regulating alternative splicing and polyadenylation (Stark et al. 2011; Grammatikakis et al. 2016; Dominguez et al. 2010). It consists of three RNA-binding domains termed quasi-RNA-recognition motifs (qRRMs) that are involved in alternative splicing, a central glycine-tyrosine-arginine-rich (GYR) auxiliary domain harboring a nuclear localization signal (NLS) thought to mediate its shuttling between the nucleus and cytoplasm, and an C-terminal glycine-tyrosine-rich (GY) auxiliary domain that may mediate protein interactions (Geuens et al. 2016; Bain et al. 2016). Interestingly, all causative variants reported so far lead to amino acid substitutions of highly conserved residues either affecting the NLS or one of the qRRMs. However, as no functional studies have been conducted, the molecular and cellular consequences of the identified variants and associated protein disturbances remain mostly unknown.

Here, we describe eight male individuals, including a pair of monozygotic twins, harboring hemizygous pathogenic variants in *HNRNPH2*. Notably, we present the first five individuals harboring nonsense or frameshift variants who display mild developmental delay, and mostly developed autistic features and psychiatric co-morbidities. We performed functional characterization of the three most common *HNRNPH2* missense variants, along with the characterization of splicing events and consecutive gene deregulation by RNA-sequencing in patient-derived fibroblasts.

## Methods

### Human subjects

All biological samples and images were obtained following written informed consent from the parents of the affected individuals. The study was performed in accordance with the Declaration of Helsinki protocols and performed in accordance with protocols approved by the Ethics Committee of the Hamburg Chamber of Physicians: PV 3802. For individuals 3, 6, 7, and 8, enrolled in a Natural History Study of HNRNP-Related Neurodevelopmental Disorder (NIH Clinical Trials.gov NCT03492060), consent was obtained in accordance with protocols approved by the Columbia University Institutional Review Board (Protocol #AAAR7203).

### Next generation sequencing

Variants in *HNRNPH2* in individuals 1–4 were identified by whole-exome sequencing or trio-whole-exome sequencing, performed according to previously described methods (Hempel et al. 2015; Lessel et al. 2018). Individuals 5–7 were referred to this study (ClinicalTrials.gov NCT03492060) as they were diagnosed with pathogenic or likely pathogenic variants in *HNRNPH2*, according to ACMG/AMP criteria, identified via clinical exome sequencing.

### Expression constructs

cDNA coding for human HNRNPH2 was obtained from Origene and subcloned into pEGFP-N3 (Clontech). Selected variants were introduced into the vectors using Quik-Change II site-directed mutagenesis kit (Agilent, Waldbronn, Germany), as previously described (Lessel et al. 2017, 2014). All constructs were verified by Sanger sequencing.

### Cell culture, transfection, immunocytochemistry, and protein immunoprecipitation and RNA-sequencing

Human embryonic kidney 293 T (HEK293T) and human bone osteosarcoma epithelial (U2OS) cells were grown on cell culture dishes and coverslips, and transfected with TurboFect transfection reagent (ThermoFisher Scientific) and Lipofectamine 2000 (Life Technology), respectively, as previously described (Lessel et al. 2017, 2014). Fixation of cells, immunocytochemical analysis, and immunoprecipitation of recombinant proteins from lysates via GFP-Trap_A (Chromotek) were performed as previously described (Lessel et al. 2017). Briefly, the protein bands were detected by chemiluminescence using a BioRad imaging system. The system was used in the “Auto” mode; this way, exposure times are optimized to maximize the signal, while avoiding saturation. The relative protein amounts of input and precipitate samples were quantified using Image Lab 6.0 (BioRad). *RNA-sequencing of primary fibroblasts.* Total RNA was extracted with the RNAeasy mini kit (Qiagen) from primary fibroblasts that were all in the same passage 8. These included primary fibroblasts of the individual 2 harboring the p.Arg114Trp *HNRNPH2* variant, three gender- and age-matched individuals affected by a neurodevelopmental disorder-not related to *HNRNPH2* mutation, and an apparently healthy male individual control 4 (GM01887 aged 7 years at sampling) obtained from the Coriell Institute. RNA-sequencing and gene expression analysis were performed as previously described (Lessel et al. 2020). Briefly, 3 μg total RNA per sample was used for library preparation with NEBNext^®^ Ultra™ RNA Library Prep Kit for Illumina^®^ (NEB, USA) and was sequenced on an Illumina Hiseq platform. Differential expression (DE) and spliceosomal defect analysis was performed using DESeq2 (Love et al. 2014). Gene ontology enrichment analysis for molecular function and biological process were obtained using PANTHER14.1 tool. spliceR package (Vitting-Seerup et al. 2014) was used for the alternative splicing analysis.

### Statistical analysis

All numerical data were imported to GraphPad Prism Version 8.0.0 and one-way ANOVA, followed by Dunnett’s multiple comparisons test, was performed by the software. *P* < 0.05 was considered to be statistically significant.

## Results

### Identification of hemizygous *HNRNPH2* variants

**Fig. 1:**
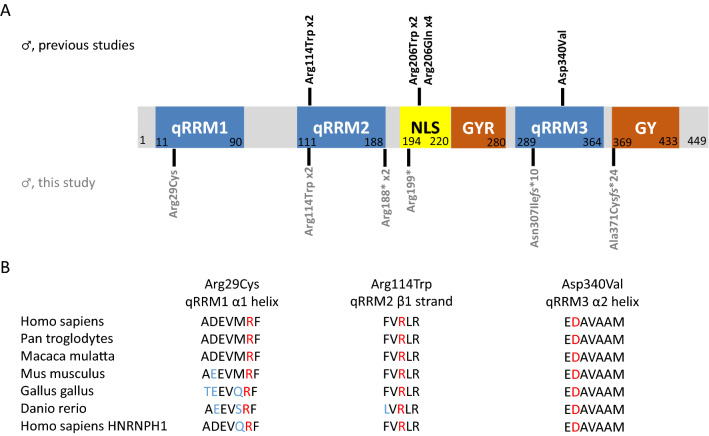
Location of identified *HNRNPH2* variants in male individuals. **A** Schematic protein structure of HNRNPH2 showing conserved domains. Quasi-RNA-recognition motifs (qRRMs) are shown in blue, a nuclear localization signal (NLS) is shown in yellow, and the central glycine–tyrosine–arginine-rich (GYR) and glycine–tyrosine-rich (GY) domains are shown in red. *HNRNPH2* variants identified in males in the previous studies are shown in black above the protein structure, and those identified in this study are shown in gray below the protein structure. **B** Evolutionary conservation of residues affected by missense mutations located within the qRRMs: p.Arg29Cys, p.Arg114Trp, and p.Asp340Val. Conserved residues are indicated in red; non-conserved amino acids are indicated in blue

We report eight individuals including a pair of monozygotic twin brothers harboring likely causative variants in *HNRNPH2* (Fig. 1A and Table 1). Two individuals harbor a previously reported de novo missense variant c.340C > T, p.(Arg114Trp) (Jepsen et al. 2019; Bain et al. 2021), with a Combined Annotation-Dependent Depletion (CADD) score of 22. CADD is a tool for scoring and quantitative prioritization of the deleteriousness of single-nucleotide variants as well as insertion and deletion variants in the human genome, which integrates a wide range of bioinformatics prediction analyses into a single measure for each variant (Kircher et al. 2014). Arg114 is a highly conserved residue within the β1-strand of the second quasi-RNA recognition motif (qRRM) (Fig. 1B). One individual harbors a novel de novo missense variant c.85C > T, p.(Arg29Cys), with a CADD score of 25.1, which affects a highly conserved residue within the α1 helix of the first qRRM (Fig. 1B). Five individuals harbor novel variants resulting in premature termination codons (PTCs) with CADD scores of 34. In more detail, individual 4 harbors the hemizygous variant c.595C > T, p.(Arg199*). As this individual was adopted as an infant, his biological parents were not available for testing and we were unable to deduce whether this variant might have been inherited. Individual 5 harbors a hemizygous variant c.918_919dupTA, p.(Asn307Ilefs*10), that was inherited from his mosaic, unaffected mother in whom this variant was identified in 9 out of 105 reads by whole-exome sequencing. Individual 6 harbors a maternally inherited c.1110dupT, p.(Ala371Cysfs*24). Finally, the monozygotic twins, individuals 7 and 8, harbor a de novo variant c.562C > T, p.(Arg188*).

**Table 1:** Clinical characteristics of individuals with *HNRNPH2* variants

Individual	1	2	3	4	5	6	7	8
Ethnicity	Caucasian	Caucasian	Caucasian	African American	Caucasian	Caucasian	Caucasian	Caucasian
Sex	Male	Male	Male	Male	Male	Male	Male	Male
Birth at (gestational week)	40	41	38	NA	38	38	36	36
Birth weight (g/SD)	3600/0.41	3955/0.48	3090/− 0.5	NA	2850/− 1.5	2810/− 1.15	2700/− 0.37	2490/− 0.84
Birth length (cm/SD)	53/0.61	50/− 1.35	52.7/0.77	NA	48/− 1	47.6/− 1.52	49/0	47/− 0.81
OFC at birth (cm/SD)	NA	34/− 1.46	41.5 (6 months)/− 1.94	NA	NA	?	33.75/0	33.5/− 0.2
Age at last examination	8 years	3 years	11 years	23 years	15 years 4 months	10 years	2 years 7 months	2 years 7 months
Weight at last examination (kg/SD)	24/− 1.01	10.4/− 2.57	25/− 2.46	72.4/0.2	62/ + 0.8	21/− 3.37	12/− 0.97	11.5/− 1.31
Length at last examination (cm/ D)	127.5/− 0.71	83.5/− 3.23	120/− 3.59	162.8/− 2.68	167.5/0	111/− 4.55	87.5/− 1.51	89/− 1.11
OFC at last examination (cm/SD)	NA (6 Years: 52/− 0.31)	45.5/− 3.91	47/− 5.15	59.6/1.71	58/ + 2	?	49.7/− 0.53	48/− 1.90
Craniofacial abnormalities								
Almond-shaped eyes	+	−	−	−	−	+	−	−
Short palpebral fissures	−	+	+	−	−	+	−	−
Short philtrum	−	−	−	−	−	+	−	−
Flat philtrum	−	−	+	−	−	−	+	+
Full lower lip	−	+	+	−	−	+	−	−
Thin upper lip	−	−	+	−	−	−	+	+
Long columella	−	−	+	−	−	−	−	−
Hypoplastic alae nasi	−	+	+	−	−	−	−	−
Depressed nasal bridge	−	−	+	−	−	−	+	+
Micrognathia	−	+	+	−	+	−	−	−
Triangular face	+	−	+	−	?	−	−	−
Pointed chin	+	+	+	−	?	−	−	−
Retroverted ears	+	−	−	−	?	−	−	−
Neurologic signs								
Intellectual disability	+	+	+	+	+	+	−	−
Motor developmental delay	−	+	+	+	+	+	+	+
Age of walking without support (months)	12	−	−	?/required some assistance till 4 years of age	18	24	24	24
Muscular hypotonia	−	+	+	+	−	+	+	+
Gait abnormalities	−	No independent walking	No independent walking		?	+	−	−
Speech impairment	+	non-verbal	Non-verbal	+	+	+	+	−
Impaired receptive language	+	+	+	+	+	+	−	−
Hyperactivity	+	−	−	+	+	+	−	−
Reduced attention	+	+	+	+	+	+	−	−
Aggressive behavior	+	−	−	+	+	+	−	−
Autistic behavior	+	−	−	+	?	+	−	−
Stereotypic hand movements	−	−	−	+	−	+	−	−
Sleep disturbance	+	+	+	+	−	+	−	−
Spasticity	−	+	−	−	−	−	−	−
Nystagmus	−	+	−	−	−	−	−	−
Strabismus	−	+	−	−	−	−	−	−
Myopia	−	+	+		−	+	−	−
Optic atrophy	−	+	−	NA	−	−	−	−
Seizures	−	+	+	+	−	+	−	−
Response to seizures treatment	−	Incomplete	Incomplete	NA	NA	+ (lamtotrigine)	NA	NA
Brain MRI abnormalities	NA	Delayed myelination, hypoplastic corpus callosum	Delayed myelination, hypoplastic corpus callosum	−(CT)	NA	−	−	−
Other abnormalities								
Scoliosis	−	−	−	−	−	−	−	−
Feeding difficulties	−	+	+	?	−	+	−	−
Contractures	−	−	−	−	−	−	−	−
Hypospadias	+	−	−	−	?	−	−	+
*HNRNPH2* variant								
Inheritance	De novo	De novo	De novo	?	Maternally (mosaic) inherited	Maternally inherited	De novo	De novo
NM_019597.4	c.85C > T	c.340C > T	c.340C > T	c.595C > T	c.918_919dupTA	c.1110dupT	c.562C > T	c.562C > T
NP_062543.1	p.Arg29Cys	p.Arg114Trp	p.Arg114Trp	p.Arg199Ter	p.Asn307Ilefs*10	p.Ala371Cysfs*24	p.Arg188Ter	p.Arg188Ter
CADD	25.1	22	22	34	34	34	34	34
ACMG classification	Likely pathogenic	Likely pathogenic	Likely pathogenic	Pathogenic	Pathogenic	Pathogenic	Pathogenic	Pathogenic
ACMG criteria	PS2, PM1, PM2, PP2	PS2, PM1, PM2, PP2	PS2, PM1, PM2, PP2	PVS1, PM2, PP3	PVS1, PM2, PP3	PVS1, PM2, PP3	PVS1, PS2, PM2, PP3	PVS1 PS2 PM2 PP3

+ , present; −, absent; *, NA, not performed; ?, unknown

*HNRNPH2* is located on chromosome Xq22.1 and contains a single coding exon, as well as one 5′-untranslated exon. Therefore, PTC variants in this gene likely escape nonsense-mediated mRNA decay (NMD) (Cusack et al. 2011), resulting in stable truncated proteins lacking at least the C-terminal GY-rich auxiliary domain.

Notably, the *HNRNPH2* variants identified here were either absent from the gnomAD dataset v2.1.1 (Karczewski et al. 2020) or observed at extremely low frequency, i.e., p.(Arg29Cys), is found in a hemizygous state only once in 183,449 alleles. Furthermore, PTC variants are completely absent from the gnomAD v2.1.1 dataset, indicating that *HNRNPH2* is extremely loss-of-function intolerant. Thus, all variants identified here were classified as either “likely pathogenic” or “pathogenic” according to The American College of Medical Genetics and Genomics (ACMG) guidelines (Richards et al. 2015) (Table 1).

### Clinical spectrum of the here identified individuals

#### Individual 1

This boy was born at term following an uncomplicated pregnancy. There were no perinatal or neonatal complications. He is the first child of unaffected, non-consanguineous parents. He has a younger brother who is unaffected. The boy was born at 40 weeks of pregnancy with unremarkable birth measurements (Table 1). His development was unremarkable until the age of 3.5 years. He began babbling around 6 months, spoke first words around 17 months, and currently, at the age of 7 years, speaks quite fluently, however not appropriate for his age. He achieved head control at 2 months, sat without support at 7 months, and walked without assistance at 12 months of age. At the age of 7 years, he was evaluated due to a suspected autism spectrum disorder. Neurologic assessment at the age of 8 years gave unremarkable results. He has difficulties with paying attention at school and shows hyperactive and impulsive behavior. Upon psychological assessment, mild intellectual disability was noted with impairment of verbal comprehension (IQ = 65), perceptual reasoning (IQ = 65), working memory (IQ = 62), and processing speed (IQ = 62) according to the Wechsler Intelligence Scale for Children (fourth edition) leading to a general IQ of 56. His testing according to the Autism Diagnostic Observation Schedule, Module 2 (ADOS-2) with a score of 8 and the Social Responsiveness Scale (SRS) with a t value of 76 revealed the diagnosis of an autism spectrum disorder.

#### Individual 2

**Fig. 2:**
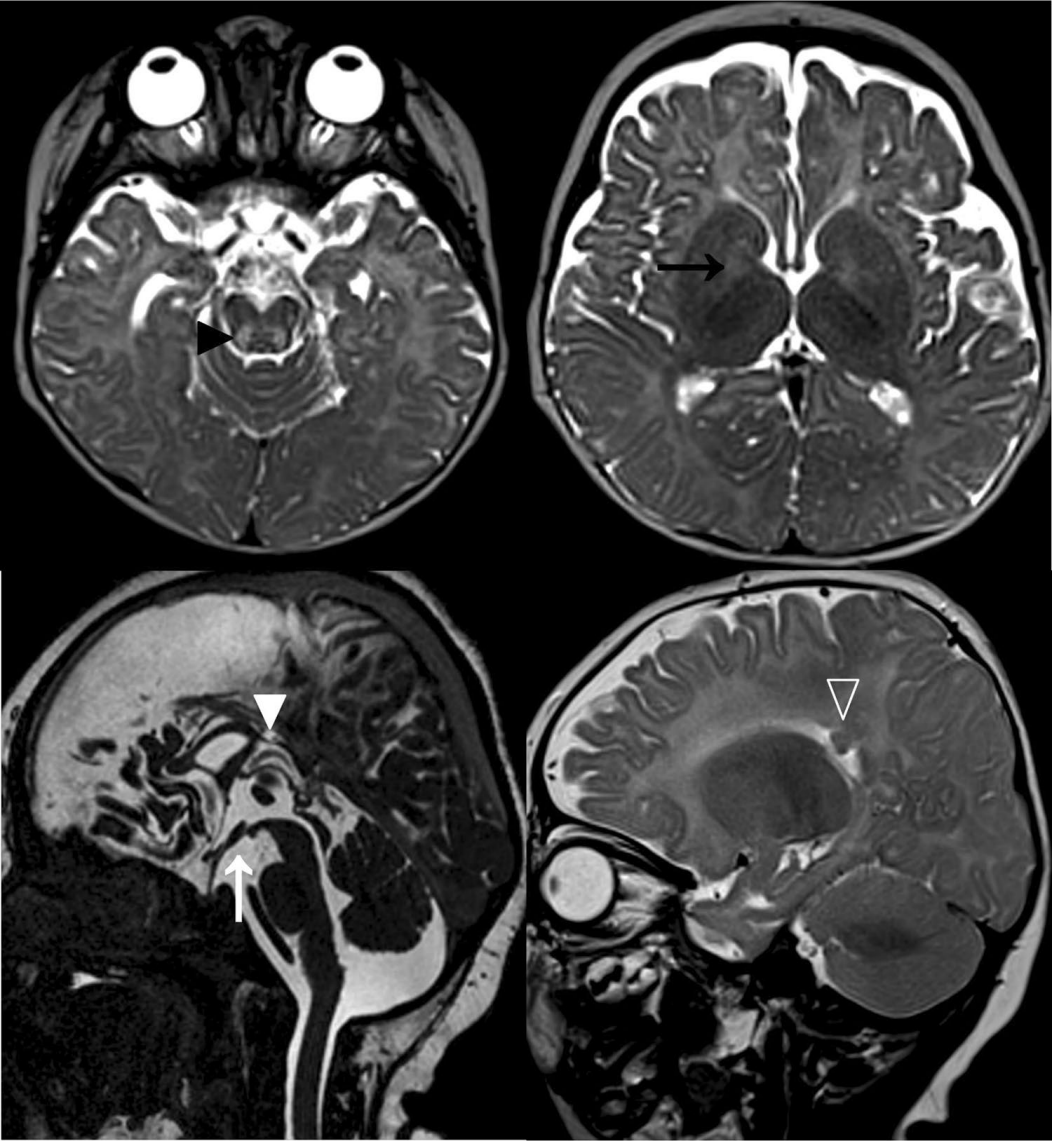
MRI findings in individual 2 at 8 months of age. Myelination appears slightly delayed for age with arborisation of the frontal white matter not yet present. Increased T2 signal intensity of the decussation of superior cerebellar peduncle (black arrow head) and medial pallidi (black arrow) is noted. At the age of 5 months, these lesions had shown restricted water diffusion, suggestive of cytotoxic edema. The body of corpus callosum is hypoplastic (white arrow head) and bilateral subependymal heterotopias are noted at this level (open arrow head). In addition, hypothalamic adhesions are present (white arrow)

This boy is the first child of unaffected, non-consanguineous parents, a 27 year old mother and a 43 year old father. The family history is unremarkable in terms of neurological or metabolic diseases. Pregnancy was uncomplicated except vaginal bleedings in the first trimester. The boy was born at 41 weeks of pregnancy with unremarkable birth measurements (Table 1) and with two natal teeth (31 and 41). From the first day on feeding difficulties and truncal hypertonia were observed. At 6 months, global developmental delay and secondary microcephaly became evident: the boy was not able to bring his hands in the middle of his body, to grasp or to roll over. He had poor visual fixation. In addition, he showed motoric restlessness. Sleep was disturbed by sleep-onset and sleep-maintenance insomnia. Extensive work-up at the age of 6 months gave unremarkable results for basic blood and extended metabolic analyses. Brain MRI showed delayed myelination, signal alterations in brain stem and to a lesser extent in basal ganglia with restricted diffusion, hypoplastic corpus callosum and cortical—intense signals alterations close to the ventricles cranial (Fig. 2). Eye examination revealed pale papillae on both eyes. At re-evaluation at the age of 9 months, we saw a restless and severely retarded infant with truncal muscular hypotonia, hypertonia of the limbs, and repeated head banging and grimacing. He did not show social interaction with only short visual contact. Motor development was severely delayed, he could not grasp, roll over, or going in the 4-point quadruped position. Weight and length were unremarkable, but the OFC was microcephalic (− 2.8 SD). By the age of 2 years, he developed therapy-refractory seizures and melatonin-resistant sleep disturbances. Extended blood, urine, and CSF analyses all gave unremarkable results. Brain MRI confirmed the previous findings. In addition, suspicion of hypothalamic adhesion, asymmetry, and inhomogeneity of hippocampi were observed. MRI spectroscopy was inconspicuous. Eye examination confirmed optic nerve atrophy and strabismus, and revealed astigmatism and myopia. At the last clinical examination at the age of 3 years, he still had a poor head control, was non-ambulatory, non-verbal, and showed severe truncal hypotonia, poor social interaction, and medically refractory epilepsy. He had a poor weight gain, with a height of 83.5 cm (− 3.23 SD), weight of 10.4 kg (− 2.57 SD), and secondary microcephaly with OFC of 45.5 cm (− 3.9 SD).

#### Individual 3

**Fig. 3:**
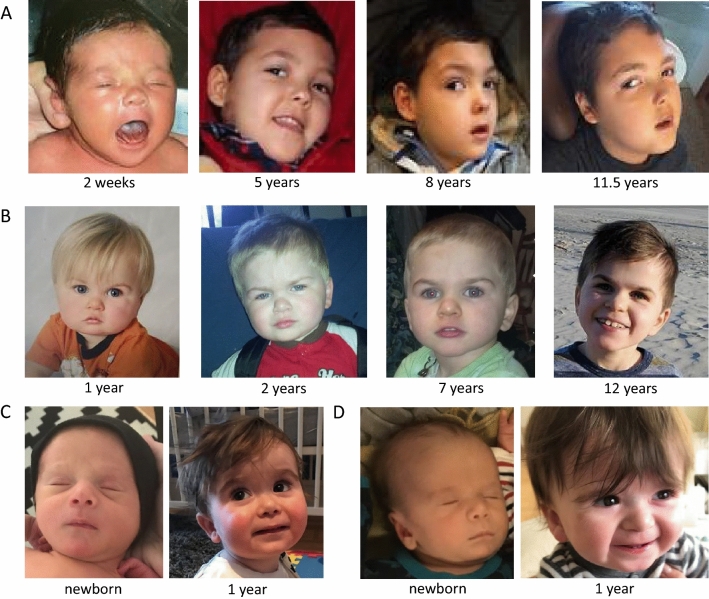
Facial images of individuals 5, 6, and 7. **A** Facial images of individual 3 at the age of 2 weeks, and 5, 8, and 11.5 years. **B** Facial images of individual 6 at the age of 1, 2, 7, and 12 years.** C** Facial images of individual 7 at birth and the age of 1 year. **D** Facial images of individual 8 at birth and the age of 1 year

This boy is the first child of unaffected, non-consanguineous parents (Fig. 3A). He was born at 38 weeks of pregnancy after an uncomplicated pregnancy. Delivery was complicated by Erb’s palsy. He had a congenital microcephaly, a truncal hypertonia, and required G-tube feeding due to severe feeding difficulties At 11 months old, he presented with clinical seizures, and his clinical semiology was described as Lennox–Gastaut Syndrome with several seizure types that were medically refractory, including myoclonic seizures daily, frequent atonic seizures, and less frequently generalized tonic clonic seizures. Serial brain MRIs revealed delayed myelination with thin corpus callosum and most recent head CT revealed diffuse brain parenchymal volume loss with ex vauco ventricular dilatation. Over the years, he developed choreiform movements and Crohn’s disease. At the last clinical examination at the age of 11 years and 6 months, he was non-ambulatory, non-verbal, showed severe truncal hypotonia, poor social interaction, and medically refractory epilepsy. He had a poor weight gain, with a height of 120 cm (− 3.59 SD), weight of 25 kg (− 2.46 SD), and microcephaly with OFC of 47 cm (− 5.15 SD). He was global delayed in all domains and subsequently diagnosed with intellectual disability. He passed away at 12 years of age from presumed sepsis.

#### Individual 4

This 23-year-old African American male was referred for a genetics evaluation for a history of intellectual disability, autistic features, and psychiatric co-morbidities including bipolar, obsessive compulsive disorder, and schizoaffective disorder. He was adopted as an infant, and adopted parents were not available to provide past medical history and no medical records were available to review. Thus, limited information is known about his past medical history. By history provided by the caregiver, there was no known report on early feeding difficulties. He was reported to have an unusual cry as an infant. Specific early developmental milestones are unknown. He is reported to have been globally delayed and required assistance walking until the age of 4 years. He was diagnosed with autism spectrum disorder and functioned below grade level in school. He required a class for children with special needs. He has not had a formal cognitive assessment as an adult and no records are available of his educational assessments. As an adult, he is verbal and able to speak in sentences and tell stories; however, his speech pattern is unusual. He uses long pauses before speaking and the topic is often disjointed from the ongoing conversation. He has basic self-help skills. He is typically very friendly but also has aggressive outbursts and obsessive behaviors. In addition to his diagnosis of ASD, in his late teens, he was diagnosed with a mental health disorder, including bipolar disorder, obsessive compulsive disorder, and schizoaffective disorder. He is followed by a psychiatrist and requires medication. He is reported to have developed seizures at 10–11 years of age and experienced a possible grand mal seizure at age 17 years. Head CT and EEG performed at that time were unremarkable. The family history is positive for a maternally related boy who has short stature, intellectual disability, unsteady gait, and limited speech. Physical examination at the age of 23 years revealed a weight of 72.4 kg (+ 0.2 SD), height of 167.5 cm (− 1.9), and head circumference of 59.6 cm (+ 1.9). No dysmorphic features were observed. Gait and balance were unremarkable.

#### Individual 5

This boy is the second child born to unaffected, non-consanguineous parents. Kidney asymmetry was noticed during pregnancy, and confirmed after birth with unremarkable renal function. Delivery occurred at term without difficulties. Birth weight was 2850 g (− 1.5 SD) and length 48 cm (− 1 SD). He walked at 18 months and spoke first words at the age of 3 years. He started to learn reading at 6 years and cognitive evaluation at the age of 9 years (WISC-IV) showed that he had no obvious intellectual disability but multiple difficulties and globally limited abilities (verbal comprehension index 66, visual spatial index 81, working memory index 70, and processing speed index 64). He developed behavioral disorder comprising social avoidance, opposition, temper tantrums, and aggressiveness. He attended a normal school with support until the age of 10 years, and then needed a class for children with special needs. The behavior disorder worsened with adolescence, so that he required treatment by loxapine and haloperidol. Psychometric testing at 14 years revealed a lower level than previously assessed (WISC-IV): verbal comprehension index 47, visual spatial index 63, working memory index 53, and processing speed index 62. Pediatric psychiatrists made a diagnosis of pervasive developmental disorder-not otherwise specified, which is currently classified as autism spectrum disorder. When examined at the age 15.5 years, he had unremarkable weight of 62 kg (+ 0.8 SD) and height of 167.5 cm (− 0.9 SD), and secondary macrocephaly with the head circumference of 58 cm (+ 2 SD). Neurological examination was unremarkable except for global slowing down of movements and speech ascribed to neuroleptic treatment. Minor dysmorphic signs were noticed including flat occiput, pointed chin, and upturned nose.

#### Individual 6

This boy (Fig. 3B) is the second child born to unaffected, non-consanguineous parents. Slow intrauterine growth was noted during the pregnancy. His mother suffered from migraines during the pregnancy, an abdominal trauma, and a severe rash requiring steroids. The mother further reports on poor fetal movements. He was born via Cesarean section due to breech position at full term. His birth weight was 2810 g (− 1.9 SD) and length was 47 cm (− 2.4 SD). During the first few weeks of life, he required heat lamp for temperature stability. He was reported to have had a mild global developmental delay. Currently, at the age of 10 years, he has several seizure types, including myotonic and absence-like, that are well controlled on lamotrigine (125 mg daily). Previous treatment with oxcarbazepine resulted in extremely aggressive behavior. He has ASD, attention deficit hyperactivity disorder, anxiety, and self-injurious behavior. Due to sleep disturbances, he received melatonin. His mother reports on recent weight loss and fatigue, difficulty swallowing, stomach pain, and poor vision. He has had a tonsillectomy and adenoidectomy for snoring and myringotomy tubes for frequent ear infections. He was wearing a hearing aid for about 2 years. He also has failure to thrive with growth hormone deficiency, his current weight is 21 kg (− 3.4 SD), and height is 111 cm (− 4.6 SD). Sensory abnormalities include decreased response to pain and increased sensitivity to temperature and touch. Brain MRI was unsuspicious.

#### Individuals 7 and 8

Monozygotic twin boys are now 2.7 years of age (Fig. 3C, D). The siblings were born full term without complications. At 3 months of age, Individual 7 was evaluated in clinical genetics and the following dysmorphisms were noted: prominent right ear lobe, depressed nasal bridge, borderline hypertelorism, thin upper lip, flat philtrum, excess nuchal skin, and right prominence on penis with deviation of penis to the left but normal meatus (no hypospadias); short 3rd and 4th right toenails, left cutaneous partial syndactyly of the 3rd and 4th toes, along with the complete syndactyly of the 5th toe. Individual 6 had plagiocephaly and gross motor delays, walking at 2 years of age. Currently, at 2 years 7 months of age, there is no significant cognitive delay, but he is being evaluated for speech therapy. No regression in skills was observed. He is left-handed and his growth parameters are within the range for his age and is normocephalic. His personality is very happy, but can have emotional episodes at times. Individual 7 also had plagiocephaly in infancy, and was also noted to have hypertelorism, cryptorchidism, and toe syndactyly. His motor delays were more notable and there was possible regression in the setting of an influenza infection. He has no previous diagnoses of autism, ADHD, or other behavioral concerns. His personality is more mellow than his brother. His medical history includes an atrial septal defect which was repaired. Both boys are socially engaged with each other and examiners. They have good eye contact with good coordination in walking, running, and throwing overhead. Neither have a maladaptive behaviors, autistic, or ADHD concerns. At last clinical examination, their weight was 12 kg (− 1 SD) and 10 kg (− 2.4 SD), respectively, height was for both 87.5 cm (− 1.5 SD), and the head circumference was 49.7 cm (− 0.5 SD) and 48 cm (− 1.9 SD), respectively (Table 1).

### *In-vitro* characterization of selected *HNRNPH2* variants

**Fig. 4:**
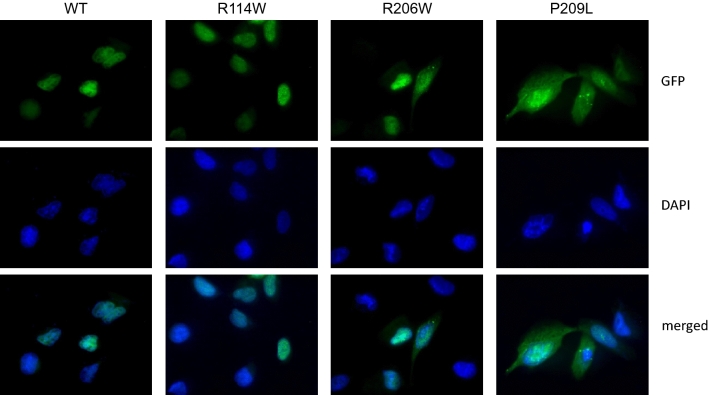
Cellular localization of selected *HNRNPH2* missense variants. U2OS cells were transfected with plasmids coding for GFP-tagged HNRNPH2 variants HNRNPH2-wt, HNRNPH2-R114W, HNRNPH2-R206W, and HNRNPH2-P209L, as indicated. Cells were fixed and stained with DAPI (blue), followed by immunofluorescence microscopy. Images were taken using 100 × magnification. Shown are representative images

To elucidate the molecular consequences underlying the differences in clinical outcomes of various *HNRNPH2* variants, we performed several lines of functional analyses. First, we investigated the subcellular localization of wild-type (wt) and selected missense variants upon expression in U2OS cells. We included the most recurrent missense variants p.Arg114Trp, p.Arg206Gln, and p.Pro209Leu. GFP-tagged HNRNPH2-wt was localized in the nucleus as expected from previous studies (Dusen et al. 2010). A similar localization pattern was observed for the p.Arg114Trp variant, located within the 2nd qRRM. In contrast, p.Arg206Gln and p.Pro209Leu, both within the NLS, were located both in the nucleus and cytoplasm, pointing to a defect in nucleocytoplasmic shuttling (Fig. 4).

**Fig. 5:**
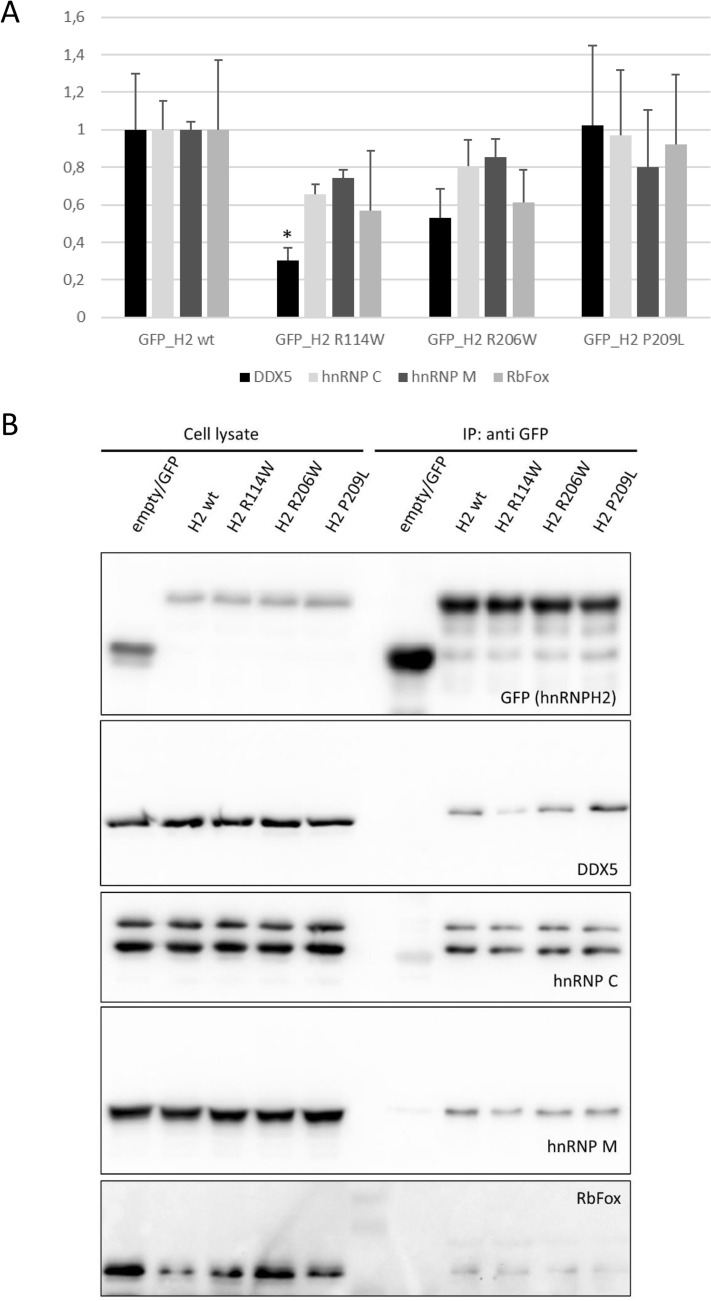
Co-immunoprecipitation analyses of selected *HNRNPH2* missense variants with components of LASR (large assembly of splicing regulators). **A** HEK293T cells transfected with plasmids coding for GFP alone, or GFP-tagged HNRNPH2 variants (wt, R114W, R206W and P209L), were lysed and analyzed by immunoprecipitation, followed by Western Blot for GFP and LASR components. Coprecipitation efficiency was determined for each interaction partner as the ratio of precipitate/input signals in Western Blots; values were normalized to those obtained with wt-HNRNPH2. *p < 0.05, ANOVA, followed by Dunnett’s multiple comparison test. **B** Representative images of immunoblots quantified in A

HNRNPH2 was reported to be a part of the large assembly of splicing regulators (LASR) (Damianov et al. 2016), a multimeric complex which regulates alternative pre-mRNA splicing (Lee and Rio 2015). Interestingly, alterations in one of the LASR components reduce the physical interaction within this complex and results in aberrant splicing (Damianov et al. 2016). Therefore, we analyzed by protein immunoprecipitation the interaction of selected *HNRNPH2* variants with LASR components DDX5, HNRNPC, HNRNPM, and RBFOX. Compared to the wt-HNRNPH2, p.Arg114Trp showed reduced interaction with the analyzed LASR components, especially a significantly reduced interaction with DDX5. The p.Arg206Gln variant showed a slight reduction in interaction with DDX5, which was, however, not statistically significant, whereas p.Pro209Leu displayed an interaction ability similar to the wt protein (Fig. 5).

### RNA-sequencing of primary fibroblasts of individual 2 harboring p.Arg114Trp

Given the previously established role of HNRNPH1 and HNRNPH2 in the regulation of alternative pre-mRNA splicing (Grammatikakis et al. 2016), the importance of alternative splicing in development and maintenance of complex neural circuits (Damianov et al. 2016), and reduced interaction of p.Arg114Trp with members of LASR, we performed RNA-sequencing in primary dermal fibroblasts derived from the affected individual harboring the p.Arg114Trp and four male control individuals. This analysis revealed 89 RNAs to be significantly upregulated, whereas 130 RNAs were significantly downregulated in the proband as compared to controls (Supplementary Table 1). Interestingly, we identified 13 upregulated and 6 downregulated RNAs transcribed from genes in which pathogenic variants have been established as the cause of a neurodevelopmental delay. These include upregulated *AARS* (Simons et al. 2015), *ATP2A2* (Sakuntabhai et al. 1999), *CAD* (Ng et al. 2015), *COL3A1* (Plancke et al. 2009), *DHCR7* (Wassif et al. 1998), *GCH1* (Furukawa et al. 1998), *GRIK2* (Motazacker et al. 2007), *MANBA* (Alkhayat et al. 1998), *MAP3K20* (Vasli et al. 2017), *MYH3* (Toydemir et al. 2006), *PC* (Carbone et al. 1998), and *RELN* (Hong et al. 2000), as well as downregulated *DHCR24* (Waterham et al. 2001), *DST* (Edvardson et al. 2012), *ECM1* (Hamada et al. 2002), *EML1* (Kielar et al. 2014), *HECW2* (Berko et al. 2017) and *MAF* (Niceta et al. 2015). Moreover, we identified 3 upregulated [*ENO3* (Comi et al. 2001), *LPIN1* (Zeharia et al. 2008) and *SCN9A* (Faber et al. 2012)] and 3 downregulated [*ATL3* (Kornak et al. 2014), *CPT1C* (Rinaldi et al. 2015), and *SGCB* (Lim et al. 1995)] RNAs whose genes have been implicated in neurological disorders.

Further analysis of the gene ontology (GO) terms linked to the differentially expressed genes revealed enrichment for terms related to development and cellular migration (Table  2). However, no GO term enrichment was identified when we specifically analyzed upregulated or downregulated RNAs.

**Table 2:** Gene ontology enrichment analysis of differentially expressed identified in primary fibroblasts of individual 2

GO biological process	Raw *P* value	FDR
Regulation of multicellular organismal process (GO:0051239)	1.77E–06	2.79E–02
Multicellular organism development (GO:0007275)	3.28E–06	2.59E–02
Regulation of cell migration (GO:0030334)	4.02E–06	2.12E–02
Anatomical structure development (GO:0048856)	4.17E–06	1.64E–02
Developmental process (GO:0032502)	5.13E–06	1.62E–02
System development (GO:0048731)	6.04E–06	1.59E–02
Positive regulation of cell migration (GO:0030335)	6.98E–06	1.57E–02
Regulation of locomotion (GO:0040012)	9.02E–06	1.78E–02
Anatomical structure formation involved in morphogenesis (GO:0048646)	9.36E–06	1.64E–02
Anatomical structure morphogenesis (GO:0009653)	9.55E–06	1.51E–02
Multicellular organismal process (GO:0032501)	1.07E–05	1.54E–02
Regulation of cell motility (GO:2000145)	1.25E–05	1.64E–02
Positive regulation of cell motility (GO:2000147)	1.29E–05	1.57E–02
Regulation of cellular component movement (GO:0051270)	1.65E–05	1.86E–02
Positive regulation of cellular component movement (GO:0051272)	1.75E–05	1.85E–02
Animal organ development (GO:0048513)	1.77E–05	1.75E–02
Positive regulation of locomotion (GO:0040017)	1.84E–05	1.71E–02
Circulatory system development (GO:0072359)	2.07E–05	1.81E–02
Positive regulation of multicellular organismal process (GO:0051240)	3.37E–05	2.80E–02
Tube morphogenesis (GO:0035239)	3.59E–05	2.83E–02
Tissue development (GO:0009888)	3.98E–05	2.99E–02
Regulation of anatomical structure morphogenesis (GO:0022603)	4.08E–05	2.92E–02
Antigen processing and presentation of endogenous peptide antigen via MHC class I (GO:0019885)	4.90E–05	3.36E–02
Embryo development (GO:0009790)	5.26E–05	3.46E–02
Semaphorin–plexin signaling pathway (GO:0071526)	6.43E–05	4.06E–02
Antigen processing and presentation of endogenous peptide antigen (GO:0002483)	7.14E–05	4.33E–02
Multicellular organismal response to stress (GO:0033555)	7.35E–05	4.30E–02
Vasculature development (GO:0001944)	7.79E–05	4.39E–02
Blood vessel morphogenesis (GO:0048514)	8.13E–05	4.42E–02

Moreover, differential alternative splicing (DAS) analysis identified 50,325 DAS events. The most common DAS event was skipped exons (SE; 36,220 events) followed by mutually exclusive exons (MXE; 4681 events), alternative 3′ splice sites (A3SS; 3763 events), retained introns (RI; 3199 events), and alternative 5′ splice sites (A5SS; 2462 events) (Table 3). This strongly suggests *HNRNPH2*-related disorders, at least those due to the p.Arg114Trp variant, to be spliceopathies on the molecular level. Notably, more than 60% of the differentially expressed genes underwent at least one DAS event (Supplementary Table 1).

**Table 3:** Summary of differential alternative splicing (DAS) analysis results performed in primary fibroblasts of individual 2

Event type	Number of events
SE-skipped exons	36,220
MXE-mutually exclusive exons	4681
A5SS-alternative 5′ splice sites	2462
A3SS-alternative 3′ splice sites	3763
RI-retained intron	3199

## Discussion

Bain-type, *HNRNPH2*-related neurodevelopmental disorder is characterized by severe global developmental delay, including motor developmental delay, severe speech, and receptive language impairment. Further common clinical signs and symptoms include growth retardation, seizures, autistic features, and psychiatric co-morbidities (Bain et al. 2021). Notably, out of the case series reported here, only individuals 2 and 3, harboring the de novo p.(Arg114Trp) within the 2nd qRRM, displayed a severe neurodevelopmental delay, similar to the previously reported individuals harboring the identical variant (Jepsen et al. 2019; Somashekar et al. 2020; Bain et al. 2021). In more detail, both individuals had a severe global developmental delay (GDD), with subsequent intellectual disability being non-verbal and non-ambulatory, severe growth retardation, truncal hypotonia, poor social interaction, delayed myelination, and hypoplastic corpus callosum, and developed a medically refractory epilepsy. In comparison, the other six individuals reported here displayed a much milder clinical course. Individual 1, harboring the de novo p.(Arg29Cys) variant within the 1st qRRM, had an unremarkable early development and was evaluated for autism spectrum disorder at the age of 7 years. He had attention deficits, hyperactive and impulsive behavior, mild ID, and impaired speech and receptive language.

Strikingly, the five individuals harboring PTC variants mostly had unremarkable early development or mild, primarily motor developmental delay. In addition, most developed several behavioral anomalies in line with autism spectrum disorder and/or psychiatric co-morbidities. Noteworthy, individual 4 inherited the p.(Asn307Ilefs*10) from his unaffected mosaic mother and individual 5 inherited the p.Ala371Cysfs*24 from his unaffected mother. These findings suggest that at least the very C-terminal PTC variants had no clinical outcome in female individuals.

Given that no functional characterization of *HNRNPH2* variants had been performed before, we analyzed the cellular localization and interaction with components of the large assembly of splicing regulators (LASR) of selected missense variants. We analyzed the two most common missense variants within the NLS, p.Arg206Gln, and p.Pro209Leu, and the most common missense variant outside the NLS, p.Arg114Trp. These experiments revealed different cellular defects. In contrast to the early assumption that p.Arg206Gln and p.Pro209Leu, localized within NLS, would not be able to enter the nucleus, we actually observed both a nuclear and cytoplasmic localization of these variants. However, none of these variants resulted in significantly reduced interaction with LASR components. Given that the wt protein is solely localized in the nucleus, we suggest nucleocytoplasmic shuttling defects (Dusen et al. 2010) as the underlying molecular consequence of these two missense variants. Comparing to NLS missense variants, the p.Arg114Trp within the 2nd qRRM resulted in nuclear localization, similar to wt, but displayed reduced interaction with a LASR components DDX5. Interestingly, despite these differences on the molecular level, the clinical spectrum of these missense variants does not seem to differ strongly.

According to the large-scale RNA-seq analysis (GTEx Analysis Release V8), *HNRNPH2* is a ubiquitously expressed gene with strongest expression in the brain (cerebellar hemisphere), whereas the expression in the skin seems to lie on the median of analyzed tissues and organs. RNA-seq analysis of primary fibroblasts of individual 2, harboring the p.Arg114Trp revealed substantial alterations in the regulation of alternative splicing. We identified a large number (50,325) of differential alternative splicing (DAS) events, which was much higher than DAS events observed in primary fibroblasts of individuals with a suspected rare mitochondrial disorder (median of 5 abnormal events per sample) (Kremer et al. 2017), along with large deregulation of gene expression. Furthermore, several genes previously associated with neurodevelopmental and neurological diseases have been identified among the deregulated RNAs, many of which underwent at least one DAS event. Albeit identified in a single HNRNPH2 affected individual, these findings strongly suggest the disorder to be a spliceopathy on the molecular level. Clearly, high-throughput RNA analyses of primary cell lines of further individuals are needed to confirm this hypothesis, and to delineate commonly deregulated RNAs and DAS events. In addition, due to the differential alternative splicing in various organs and tissues (Nilsen and Graveley 2010), it would be extremely important to also perform similar analyses in iPSC-derived neurons from affected individuals. Commonly deregulated genes in these cell lines might represent potential targets for therapy. Notably, such analyses require extensive further work and should be the main aim of future studies dealing with functional characterization of *HNRNPH2*-related disorders.

Furthermore, it remains unknown why PTC variants in *HNRNPH2* result in a rather milder clinical outcome as compared to the missense variants, especially those located within the NLS and the p.Arg114Trp mutant. At this point, we can only speculate that HNRNPH1, which shows a strong homology (96%) to HNRNPH2, and whose function might therefore overlap (Grammatikakis et al. 2016) could compensate for the loss of HNRNPH2.

In conclusion, we further expand the clinical spectrum of *HNRNPH2*-related disorders, provide first molecular evidence for pathogenicity of selected *HNRNPH2* missense variants, and suggest the disorder to be a spliceopathy on the molecular level.

## Supplementary Information

Below is the link to the electronic supplementary material.

Supplementary file1 (XLSX 41 KB)
